# Population health management of human phenotype ontology

**DOI:** 10.3389/frai.2025.1496935

**Published:** 2025-08-13

**Authors:** James Andrew Henry

**Affiliations:** Institute of Biomedical Sciences, London, United Kingdom

**Keywords:** population health management (PHM), human phenotype ontology (HPO), biological modelling (BM), predictive health, precision care, classifications, international standards, artificial intelligence

## Abstract

**Aims:**

Population Health Management (PHM), through strategic integration of the Human Phenotype Ontology (HPO), emphasises the responsible use of digital infrastructure and comprehensive genomic data to promote good health and wellbeing. The UK seeks to steward medical science and phenotype practices in primary care settings with technical approaches for developing a national Biological Modelling (BM) ecosystem. By recognising diverse global healthcare systems, this manuscript offers a means for nations to adapt their HPO operational deployment for global PHM harmony.

**Methods:**

The methodological approach incorporates primary care services and funding assessments to address digital infrastructure needs, ensuring secure national data access. Evaluations include ISO standards, systems thinking, alignment of UK infrastructure with informatics requirements, and AI norms within the ecosystem. Specific use cases for genomic predictive health pre-eXams and precise care eXams are assessed, alongside strategies for bias mitigation to ensure fairness in AI-driven classifications.

**Recommendations:**

The manuscript advocates for establishing local agile ecosystem groups for PHM, regional Higher Expert Medical Science Safety (HEMSS) stewardship, national HPO value-based care models, and integrating global PHM general intelligence. Real-world AI and clinical practice comparisons are emphasised for validating digital twin personalised BM via Gen AI in the HPO transformation ecosystem.

**Discussion:**

Federated Learning and GPT-5 technologies advance international PHM by supporting HPO transformations. Standard personalised BM learning addresses intranational HPO variances, requiring individual classifications. National HPO roadmaps prioritise inclusiveness and stakeholder engagement, supported by informed consent and quantum intelligence. Ethical and equitable HPO deployment demands proactive stewardship and national cooperation to address limitations and ensure robust classifications.

**Conclusion:**

Unified, data-driven HPO transformation utilising advanced AI and genomics is essential for personalised healthcare delivery. Rigorous assessments, ethical considerations, and global collaboration enable impactful implementation. National PHM ecosystems guided by HPO transformation in classifications sustain healthcare, advancing patient outcomes through responsible innovation and informed policy development.

## 1 Introduction to human phenotype ontology transformation

Human Phenotype Ontology (HPO) transformation represents an innovative approach to risk stratification in Biological Modelling (BM), mapping a standardised vocabulary for disease predictors and intercepts (Putman et al., 2023; Yang et al., 2021). The UK government envisions HPO as the foundation of primary care, aligning with its science and technology objectives and promoting international collaborations as a force for good (GOV.UK, 2025a).

This manuscript builds upon United Nations initiatives and global goals to advance Population Health Management (PHM) through AI-powered genomic health infrastructure, stewarding personalised practices (World Health Organization, 2023a; Global Alliance for Genomics and Health, 2017). By enabling a uniform digital healthcare strategy, HPO transformation progresses internationally in a real world of consistency in predictive health and precision care classifications (World Health Organization, 2021).

**Figure 1:**
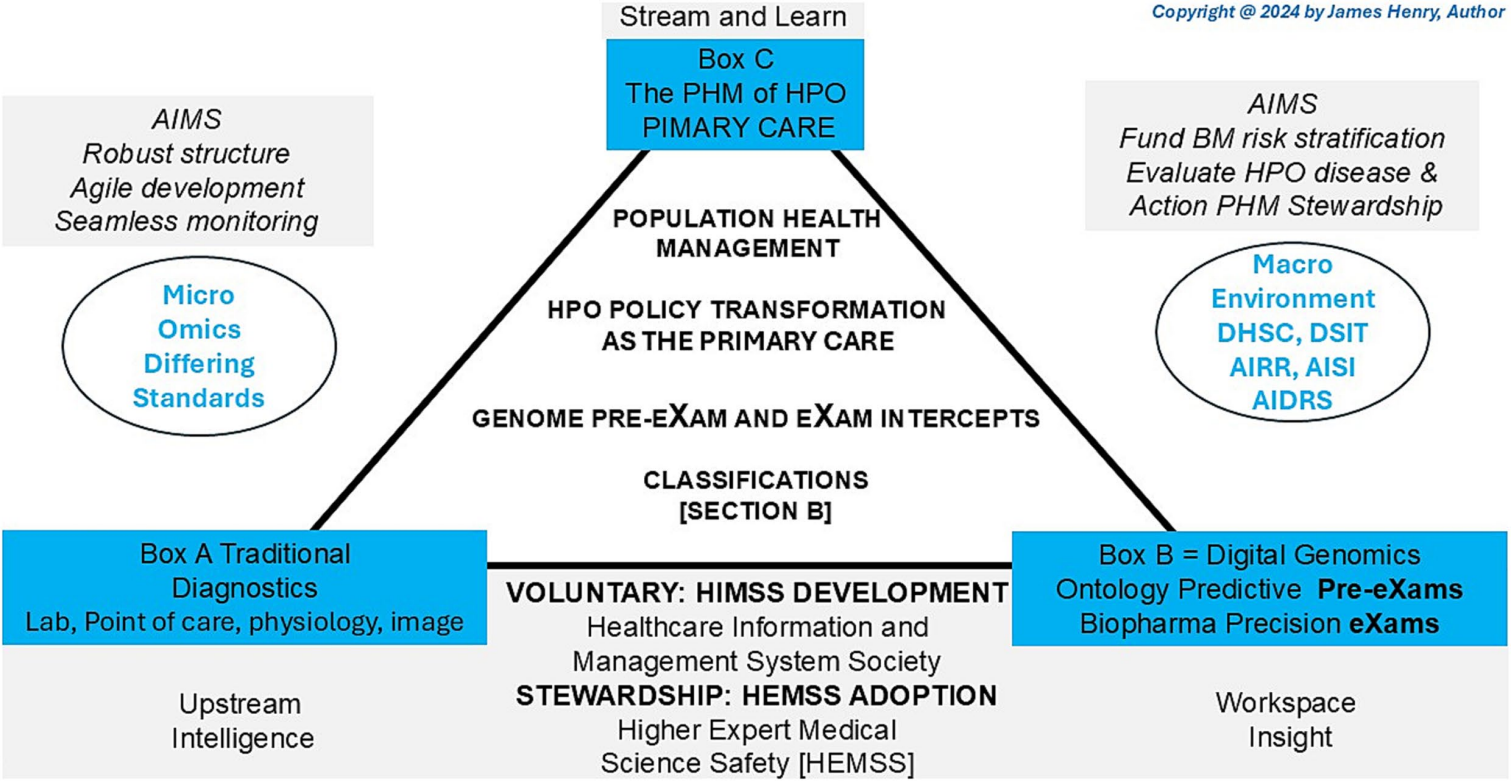
Population health management, the HPO transformation in traditional diagnostics.

HPO digital stewardship establishes a standardised ecosystem that integrates genomic and socio-environmental factors, delivering socioeconomic benefits and value-based care (Organization for Economic Co-operation and Development, 2025). As illustrated in Figure 1, PHM uses digital tools in data diagnostic settings, reshaping primary care medicine with generative AI-powered classifiers that drive meaningful transformations across a lifetime of Biological Modelling (BM).

### 1.1 Background to HPO classifications and stewardship

Figure 1 depicts the HPO classification, illustrating traditional diagnostics (Box A) and digital classifications (Box B) to reform conventional primary care medicine (Box C) with BM predictor pre-eXams and precise care eXam intercepts. From 2014 to 2017, the UK NHS and the author brought reports integrating scientific assurance into hospital governance to enhance HPO (NHS England, 2014; Henry, 2017). By 2018–2021, medical interoperable engagement and digital patient inclusivity achieved growing global recognition in advisory IT organisations (Centered for Connected Medicine, 2019; Global Digital Health, 2021). From 2022 to 2024, pangenome research and global biobanks would align for the author to propose HPO transformation in the PHM of BM classifiers through science and technology (National Human Genome Research Institute, 2023; Mordor Intelligence, 2025).

Figure 1 illustrates the 2025 Higher Expert Medical Science Safety (HEMSS) proposal, which aims to bridge HIMSS with hybrid AI practitioners and national Biological Modelling (BM) transformations. This proposal evaluates and implements the adoption of HPO, supported by organisations such as pathology institutes and medical colleges with accreditation and governance bodies. These entities advance science and technology by ensuring quality data and digital competence while addressing variations caused by competing informatics features (Royal College of Pathologists, 2022; Haleem et al., 2022). The author examines ISO 15189:2022 clauses, HIMSS levels, and ISO/IEC JTC 1/SC 42 norms to establish an HPO “safe space” for public health, patient safety, and biopharma outcomes through Gen AI classifiers, reducing variation in pre-eXam and eXam practices (ISO, 2022; Healthcare Information and Management Systems Society, n.d.; International Organization for Standardisation, 2017).

### 1.2 Overview of HPO transformation

Section 2 introduces the manuscript’s aims, highlighting the HPO transformation through standardised medical science laboratories and national tools that establish a safe space for Biological Modelling (BM). Section 3 evaluates the PHM ecosystem by analysing HIMSS levels, AI standards, HPO transformation classifications, and assessments of resources, controls, and stewardship. Section 4 outlines strategies for HPO transformation, focussing on value-based care initiatives and integrating real-world AI validations to improve healthcare delivery. Section 5 explores international, national, and federal dimensions, examining technology reform, classifier metrics, and limitations within the HPO ecosystem. Section 6 concludes with key findings, presenting a unified strategy for HPO transformation that benchmarks innovation for PHM sustainability.

### 1.3 Classification of HPO transformation approaches

The manuscript stewardship of classical Genomic Predictive Pre-eXams in genetic evaluations of single-cell or germline WGS for BM is progressive. Precise eXam Intercept oversight represents a lifetime of accurate biopharma mitigating disease within the HPO transformation. These processes for PHM are developed using X (Gen AI) resources, evaluation, and regulation. Following a period of stewardship to assess predictors and intercepts, these are adopted for HPO primary care. Fit-for-purpose X is achieved through an ecosystem to ensure fairness, explainability, security, and trustworthiness in PHM.

## 2 The aims for human phenotype ontology transformation

**Figure 2:**
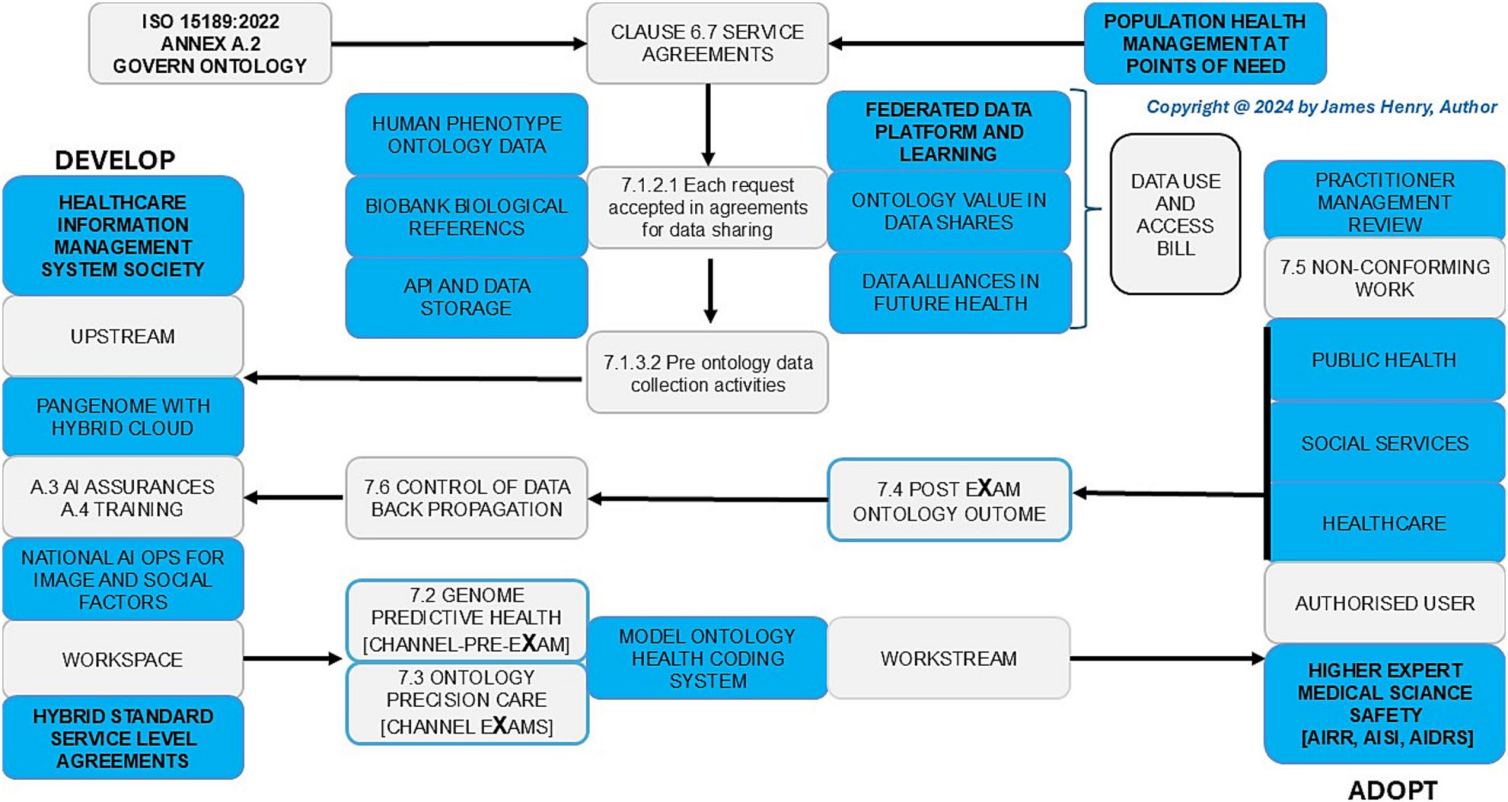
Population health management, the HPO transformations agreements: point of care (grey) and point of need (blue in safe space).

**Figure 3:**
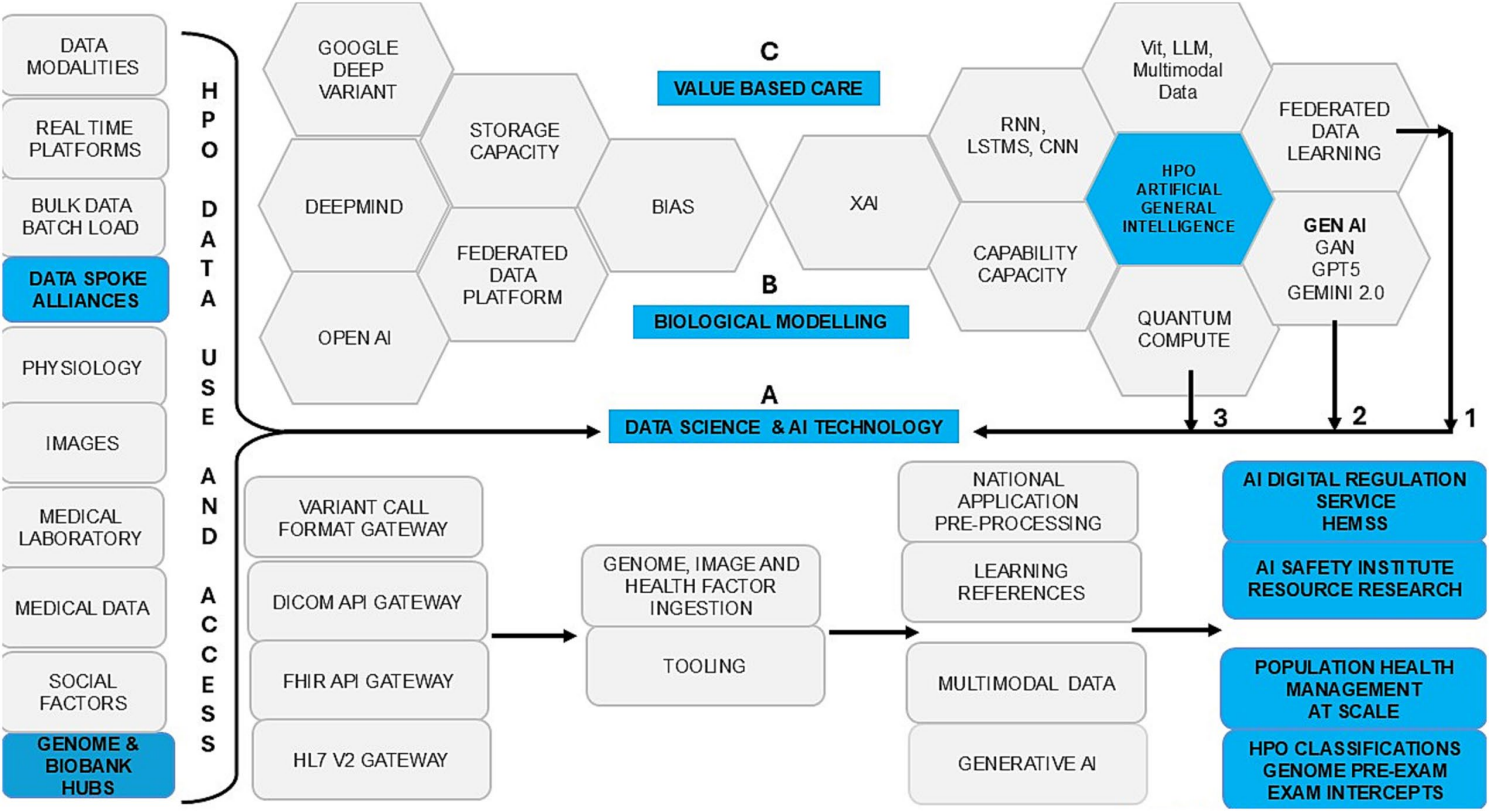
Population health management, HPO transformation with science and technology.

Figure 1 shows HPO transformation with HIMSS maturity and capacity with HEMSS principals for PHM and BM classification capabilities through standards and tools. In Figure 2, the multiple international agreements for HPO transformation aim to arrange AI Laboratories through HIMSS maturity. Figure 3 shows the UK vision for HPO transformation that cluster AI resources and safety evaluation with stewardship. The HPO mission differs among nations, with options in Section 2.1 Standard medical science laboratory resources and processes, Section 2.2 Standard ICB technical approaches to population health, and Section 2.3 National PHM of HPO transformation as standard.

### 2.1 Standard medical science laboratory approach to resource and process

In Table 1, ISO 15189:2022 clause requirements for medical laboratory quality and competence are subject to justified variation for digital HPO transformation with safe space BM (The Association of Clinical Biochemistry and Laboratory Medicine, 2023). Nations aim for a standard HPO medical science laboratory approach, necessitating arrangements with HIMSS maturity for PHM in an AI digital environment at the point of need (view Annex A of ISO 15189:2022 to integrate AI governance, training and assurance as proposed in the authors unpublished work from 2014) (The Association of Clinical Biochemistry and Laboratory Medicine, 2023). Thus, medical science BM resources (Clause 6) for predictor and intercept HPO control (Clause 7) can align AI infrastructure and governance (Clause 5) with management system requirements (Clause 8).

**Table 1:** Medical laboratory requirements integrate for national AI laboratory arrangement.

Clause	Document contents	Justified variation	International HPO transformation
1	Scope	HPO transformation	Global
2	Normative reference	Genome predictor pre-eXam Biopharma eXam intercepts	International classifications
5	Structure and governance	PHM structure and governance	HIMSS/HEMSS
6	Resource requirements	HPO transformation	Quality data
7	Process requirements	Biological modelling	Digital competence
8	Management systems	Ecosystemic PHM	OSEIPS
Annex A	Point-of-care agreements (view Figure 2)	Point of need adoption (view Tables 2, 3)	National stewards (view Figure 3)

HIMSS, Higher Information Management System Society, Higher Expert Medical Science Safety, OSEIPS Ontology System Engineering Initiatives for Patient (Public) Safety.

### 2.2 Standard integrated care board technical approach to population health

As tabled, the UK Health & Care Act 2022 mandates ICBs to establish a technical approach with robust quality data resources and agile digital process control for seamless BM in an HPO transformation for PHM (UK Parliament, 2022).

1. Table 1 shows that the ICB transformation aligns with ISO 15189:2022 Annex A for AI governance, training, and assurance (ISO-International Organization for Standardization, 2022).
2. Table 2 shows the ICB aims for HIMSS maturity to drive AI risk HPO stratification and disease segmentation (NHS England, 2021a).
3. Table 3 shows BM system thinking for HPO genomic transformation in trusted pre-eXams and eXam intercept research (Graham et al., 2022).

**Table 2:** HIMSS development of HPO transformation and HEMSS PHM adoption.

HIMSS	Population health management
One	Automate: e.g., laboratory, radiology and pharmacy information & management *Expand for all genomics, image and health factors*
Two	Clinical data repository information *Expand for PHM implementations*
Three	Clinical decision support tools *Plan tools to predict health in BM*
Four	Computerised provider order entry for medications *Plans tools for precise care in BM*
Five	Closed-loop medication administration *Evidence pharmacogenomic in the BM loop*
Six	Physician documentation *Evidence scientific themes to record HPO predictors and intercepts*
Seven	ECOSYSTEM: HIMSS development – HEMSS adoption *Population health ontology records with workforce inclusivity*

**Table 3:** PHM ecosystem thinking.

Personalised system thinking	Population health management human phenotype ontology safe space
HPO classification experts	PHM ecosystem for certified practice in hybrid
Point of need-local rational	Medical genome and social health science PHM goals
National fair & just culture	Institutes & individuals’ openness, trust & fairness
PHM demand & pressure	Utilisation of resources in future HPO initiatives
PHM resource constraints	Increase or restrict the ability to meet HPO demand
PHM interactions & flows	Activities relate & interact citizen-centric cycles
HPO trade-offs	Resolve conflict for ontology activities & management
AI performance variability	Understand variance in practices, omics and images
PHM emergence	Indefinite predictors & intercepts arise
Ontology equivalence	HPO needs evidence work as imagined

Rows (principals): 1–3 represent the view of people; 4–5 represent PHM conditions and context that affect HPO work; 6–8 concern persons in flow; 9–10 Personalised practice for ontology system predictive health and precise care.

Figure 1 underscores a decade of infrastructure for HIMSS maturity in a proposal that aims for Higher Expert Medical Science Safety (HEMSS) in a national stewardship of agile HPO transformations in seamless PHM. HPO transformation integrates genomic and multi-data resources with technical oversight of quantum federated learning for advanced AI (Deshprabhu, 2023; Dhade and Shirke, 2024). National ICB capacity with AI medical laboratories unlocks high interoperable speeds that drive use case validation in lifecycles with standard nomenclature for predictors and intercepts, a classification (Genomics England, 2021; Our Future Health and NHS, 2022).

### 2.3 National PHM of HPO transformation, as standard

Figure 1 illustrates the operational framework for HPO transformation, envisioned as a joint aim of the Department for Science, Innovation and Technology (DSIT) and the Department of Health and Social Care (DHSC). The strategy focusses on BM risk stratification, HPO disease evaluation, and PHM stewardship proposals to address emerging healthcare opportunities (Department of Science, Innovation and Technology, 2023; GOV.UK, 2025b). The biobank and life science approach uses genome, social factors, and images to enhance the PHM of HPO transformation with biopharma (Biobank, 2025; Pharmaphorum, 2025). Three pivotal entities support this effort:

1. The UK AI Research Resource (AIRR) drives genomic and AI healthcare to advance the classifications (UK Research and Innovation, 2023a).
2. The AI Safety Institute (AISI) ensures AI development while safeguarding public trust in predictive health and precise care (GOV.UK, 2023a).
3. The AI Digital Regulation Service (AIDRS) develops HPO pre-eXams and eXams for adoption by ICB organisations (NHS, 2025).

Through the combined leadership of DHSC, DSIT, AIRR, AISI, and AIDRS, this stewardship of science, innovation, technology, resource, evaluation, and regulation of HPO transformation position the UK as a global leader in AI-driven PHM (Department of Science, Innovation and Technology, 2023; GOV.UK, 2025b; Biobank, 2025; Pharmaphorum, 2025; UK Research and Innovation, 2023a; GOV.UK, 2023a; NHS, 2025).

## 3 Assessment of human phenotype ontology transformation

The assessment of HPO transformation reviews PHM resources, process control, and agile group developments to evaluate and advance risk stratification and disease segmentation as a digital norm in hybrid AI stewardship, data training, and analytic assurance of PHM resources in Figure 2. The multifaceted agreements and stewardship are explored in Section 3.1, Analysis of PHM resources, Section 3.2, Evaluation of HPO process control, and Section 3.3, Investigation of BM through classification metrics.

### 3.1 Assess PHM ecosystem resources

A PHM ecosystem for HPO transforms research resource funds and data assets in a reform to conventional primary care with the classification of BM using predictors and intercepts. Subsection 3.1.1 assesses primary care services and funding, 3.1.2 assesses data points for digital needs, and 3.1.3 assesses stewardship for national data access and use.

#### 3.1.1 Primary care services and funding

PHM support for primary care services is derived from the Health and Social Care Act (HSCA) 2022, compliance with fact sheets and practice codes covering doctor training, conflict resolution, technical builds, record management, and data communication (GOV.UK, 2021). The NHSE Innovation Ecosystem Review Programme represented a key initiative within ICB finance and innovation, directing funding to distribute resources for health education and PHM through biobanks (NHS, 2023; NHS Health Education England, 2020). Various funding initiatives, such as the Cancer Drugs Fund, the Innovative Medicines Fund, and the Med-Tech Funding Mandate, enhance the adoption of medical technology and innovative medicines while ensuring cost-saving diagnostics, analytics, and digital products receive adequate investment (NHS Health Education England, 2020; NHSE England, n.d.). A future funding initiative for cluster analysis within the HPO transformation aligns with DHSC/DSIT resources, evaluations, and regulatory frameworks (Department of Science, Innovation and Technology, 2023; GOV.UK, 2025b; Biobank, 2025; Pharmaphorum, 2025; UK Research and Innovation, 2023a; GOV.UK, 2023a; NHS, 2025).

#### 3.1.2 Assess data points for digital need

Ecosystem resources for genomics and point-of-care medical devices use data asset exchanges across points of need (Table 1 Clause 6 and Annex A). The justified standard variation of ISO 15189:2022, combined with HIMSS for continuous APIs, deploys the PHM ecosystem for HPO transformation (Splunk, 2022; Royal College of Pathologists, 2022). Assessments of data points for digital classifier needs include the following:

1. Sufficient developers and capacity to perform AI with robust architecture and algorithms for use case insights to scope continual education for staff professional development with ML/DL from local need to national oversight.
2. HPO prediction and precision with data alliances that support users in accessing and streaming analytics projects through organisational nodes across federated data platforms.
3. Digital equipment arranges for identifiable management, DevOps, use cases, instructions for use, contingency, adverse incident reporting, logs, and workspace storage records.
4. Equipment assurance for data mining, ML training, or NAS XAI provides trust in predictive and precision analytic methods with meteorological traceability, as appropriate, while tracing processes for HPO monitoring.
5. Evaluation of AI backpropagation methodologies as a process that recalibrates its nodes for greater accuracy in workstream outputs, with continual improvement in citizen monitoring.
6. Meteorological comparatives, as required, may be relevant within some assurance systems, such as XAI, which require hybrid arrangements specific to quality assurance arrangements.
7. Informatics is an asset consumable feature for predictive health and precision care that improves each ontology system’s analytics selection, acceptance testing, and management through biobank workspace and workstreams.
8. Digital experts’ services in higher medical sciences for safety open the genome networks, national imaging, and social factors as cooperatives, which serve as a point of need to provide ontology evaluation and monitoring of an intercept.
9. Service agreements from medical (research) to social welfare as data alliances cooperate with health practitioners and the community through partnerships that expedite expert projects.
10. Civic cooperatives for populace analytics scale up for continual and batch data integration as AI use case agreements develop PHM projects for adoption and user-defined access.

#### 3.1.3 Stewardship for national data access and use

In Figure 2, the data (use and access) bill aims for wellbeing and growth in support of a modern digital government that transforms people’s lives with HPO aligned research and development evaluations for routine predictive health pre-eXams and precision care eXams. In Table 2, HEMSS stewards of HIMSS ensure DUAB requirements integrate national biobanks, UK rare disease strategies, major condition plans, and biopharma actions to impact generations in future health (Tech UK, 2024; Genomics England, 2024; Our Future Health, 2021). HPO stewardship makes citizen data easily transferable, facilitating time and improving health outcomes at each point of need (The Association of Clinical Biochemistry and Laboratory Medicine, 2023).

In Figure 3, data access and use are bidirectional with robust GPT-5, agile quantum developments, and seamless federated learning for HPO risk stratification and pathology segmentation in value-based care plans. Stewardship ensures PHM adoption as the Information Commission asserts powers for responsible and ethical data use in an HPO transformation, which enhances public trust in digital health initiatives (Information Commissioner's Office, 2024). The AIDRS of PHM developers and BM adopters of research need steward HPO predictors and lifetime intercepts from science themes (NHS Health Research Authority, 2023; Pharaphorum, 2025).

### 3.2 Evaluates HPO process control

Table 3 shows ecosystem HPO thinking for BM points of need to mitigate data storage and compute capacity costs with classifier capability. Figure 2 reviews agreements for HIMSS/HEMSS, the point of care, and the point of need. Figure 3 reviews HPO process control for predictor developers and intercept adopters of AI. Subsection 3.2.1 assesses HPO activities, 3.2.2 evaluates HIMSS level assent, and 3.2.3 assesses AI norms in a new frontier for an ecosystem.

#### 3.2.1 Assess HPO activities

From Table 1, Clause 7, the PHM framework of HPO ensures safe process control by aligning Box B for the classification of physical and mental healthcare with digital competence. Figure 1 shows PHM triangulation and justifies HPO transformation in public health, patient safety, and equity classifiers. ISO 15189:2022 activities are categorised as norm references in variations of Sections 7.2, 7.2.3, and 7.4 as follows (Royal College of Pathologists, 2022):

1. Pre-eXams—Patient or sample pre-examinations (Box A) are transformed into digital health pre-eXams, predicting HPO from DNA donations (Box B). PHM agile groups develop personalised user insights from genomic information. Intelligence oversight, assurance, and training are embedded as standardised practices (Table 1; Annex A), with HIMSS (Table 2) and ecosystem thinking (Table 3) (Royal College of Pathologists, 2022; Henry, 2017).
2. The eXams—Activities include phenomics, imaging, physiological, and health factors (Box A), which evolve into digital precision care eXam classifiers (Box B). Digital Regulation Services ensure greater public confidence in a PHM ecosystem using AI oversight from trained and assured programmes, driving improved health outcomes (Tables 1–3) (Royal College of Pathologists, 2022; Henry, 2017).
3. Post-eXams—Post-examination processes providing clinical significance (Box A) are refined as digital post-eXams (Box B), delivering personalised outcomes monitored across conventional medicine practices (Box C). The PHM era integrates traditional diagnostics and medicine with accurate outcomes achieved, while backpropagation ensures HPO reliability for predictors and intercepts (Royal College of Pathologists, 2022; Henry, 2017).

Classification of BM predictive genomic pre-eXams and precise care eXams intercept requires resource, evaluation, and regulation of HPO transformation activities (UK Research and Innovation, 2023a; GOV.UK, 2023a; NHS, 2025).

#### 3.2.2 Evaluate UK infrastructure and HIMSS level assent

Table 2 presents the UK Infrastructure to invest in the next decade with consistent finance expenditure for PHM projects over short- and long-term business planning, enabling skills, technologies, and practices (NHSE, 2024). National Infrastructure Assessment from the National Infrastructure Commission should assess HPO requirements with recommendations to address them (GOV.UK, 2024a). In Table 2, HIMSS analytics maturity supports AMRAM, IFRAM, CCMM, and EMRAM, which improve PHM in HPO assents that realise BM classifications (HIMSS, 2018).

Table 2 outlines maturity levels in HIMSS initiatives for the PHM of HPO in BM (HIMSS, 2024). Meanwhile it is proposed that HEMSS principles as detailed in section 4.2 steward the adoption of the following:

1. The integration of Generative AI (Gen AI) enables BM classifications that predict health (Pre-eXam) and deliver precision care (eXam).
2. Explainable AI (XAI) ensures transparency and accountability in HPO decision-making processes.
3. Bias mitigation, with equitable and inclusive practices to enhance PHM run before and after HPO performance checks.

#### 3.2.3 AI norms in a new frontier for an ecosystem

Table 2 ISO AI norms open PHM frontiers for HPO in a personalised health and social care ecosystem of hybrid partnerships (International Organization for Standardisation, 2018). As digital activities predict health and precision care, they demonstrate conformance to IT-AI/ML/DL concepts, terms, taxonomy, and transparency to process life cycles from big data analytics (ISO/IEC 5338, 2023). Structuring knowledge in digital HPO is a mega-data process in architecture with AI application to engineer PHM (ISO/IEC 5392, 2024).

Gen AI management and computing require validation and verification, while ML computing treats bias in classification-related tasks (ISO/IEC TS 12791, 2024). Norms span use cases and the controllability of AI systems which develop quality data for analytics through analysis, measures, management, process, and stewardship, with neural networks and transformative HPO robustness assessed (ISO/IEC 24029-2, 2023). Nationally, ISO 15189:2022 Annex A engages HIMSS with ISO/IEC JTC1/SC 42 and conformance to:

1. ISO/IEC 38507:2022—An oversight of the implications of organisations using AI to guide members on expert use of ontology insight. The guide for effective and efficient organisational AI expands on a national ecosystem of ontology national intelligence.
2. ISO/IEC TR 24368:2022—An overview of ethical and societal concerns opens public AI challenges for regulators, interest groups, and technologists as the information relates to principles, methods, and an overarching PHM ecosystem.
3. ISO/IEC 24668:2022—Process management for the big data analytics framework to develop analytics across sectors and specify access management for informatics to include process categories and their interoperability.
4. ISO/IEC TR 24372:2021—Computation ecosystem approaches provide overviews of excellence in AI HPO approaches and describe the main characteristics, algorithms, and activities used.
5. ISO/IEC TS 4213:2022—Assessment of ML classifies performance and specifies methods for measuring the performance of models, systems, and algorithms. It is one of many ISO standards that meet sustainable goals.
6. BS 30440:2023 framework AI is used in health to assess technology developers and validate products. Health providers can mandate vendor products be certified to assure themselves and service users that the algorithm is effective, fair, and safe.
7. ISO/IEC TR 24027:2021—Bias in AI systems that aid decision-making evaluate techniques to measure and assess biases and address vulnerabilities in a lifecycle as what appears technical is indeed in the interest of each ontology system’s biological and health factor analysis.

Table 3 shows PHM ecosystem thinking as HPO medicine elevates AI for continuous BM improvements in WGS predictive health pre-eXams and precise eXam intercepts.

### 3.3 Analyses BM through classification metrics

In Figure 3, BM data exchanges realise classifiers in HPO transformation in an ecosystem of PHM capabilities with metrics for value-based care. Disparity in national PHM, conventional medicine, and traditional diagnostics is with differing Genomics QA, HPO metrics, and libraries (AI Safety Institute, 2024). AI infrastructure evidence predictors and biopharma from BM research in AI Safety Institute clusters with GMS and Biobanks metrics to evidence standard classifications (GENQA, 2023; Observational Health Data Science and Informatics, 2022). Section 3.3.1 evaluates use cases in genome predictive health pre-eXams. Section 3.3.2 evaluates use cases for precise care eXams. Section 3.3.3 enhances equity through bias mitigation in AI-driven classifiers.

#### 3.3.1 Evaluate use cases in genome predictive health pre-eXams

Table 4 shows use case support for genomic predictive health pre-eXams as BM assesses HPO evidence in medical primary care reform and disease prevention through germline and single-cell analysis with predictor metrics in advanced AI, which improves personalisation.

**Table 4:** Science and technology metrics for genomic predictive health pre-eXams.

Science aspect	Technological methods/tools	Key metrics and outcomes
Alignment	BWA-MEM, Minimap	High-accuracy map of the nucleotide read to reference pangenome
Variant calling	DeepVariant, GATK, DRAGEN	DV > 99 SNP and INDEL accuracy. DRAGEN-x30 faster pipelines
Functional annotation	FAVOR GTP, GENOME GTP, ANNOVAR	Pre-eXam insight by analysing gene function, occurrence rates, and disease
Integration	Combe short (Illumina) & long read (PacBio, Oxford Nanopore)	Improves structural variant detection rates by up to 20%
AI-driven analysis	DNABERT, Sention	Advances classification and structural variant analysis via precise AI models

WGS pre-eXams assess BM accuracy, efficiency, and clinical utility for rare diseases, demonstrating increased diagnostic yield, while pilot studies emphasise the impact on clinical and patient management and even time to diagnosis (Smedley et al., 2021). GMS programmes integrate omics testing into routine care in common major conditions, tracking HPO diseases in pre-eXams (NHS England, 2017). The interpretation of sequence variants relies heavily on established guidelines, which use sensitivity and specificity to determine pathogenicity as we expand on conditions for early action (Ziegler et al., 2024). International collaborations, such as those facilitated by GA4GH, are crucial for data sharing and standardisation, with metrics consistently applied across diverse research and healthcare settings (Rehm et al., 2021).

Exploring GPT-5 impact on pre-eXams/eXams via multimodal data analysis for personal care annotate genetic variants with social factors using resources such as FAVOR to simplify insights (Quazi et al., 2024a). Predictive BM powered by GPT-5 enhances disease forecasts by integrating comprehensive evaluations and monitoring solutions, ensuring accurate and reliable opportunities in an OpenAI preparedness framework (OpenAI, n.d.). A study in which GPT-4 achieved an F1 score of 0.96 for disease progression expects greater accuracy, sensitivity, and specificity metrics from GPT-5 PHM with bias mitigation (Innovate healthcare Health Imaging, 2023; Richards et al., 2015). HPO reasoning with synthetic data is further expected to improve classifier accuracy (Berman, 2024).

#### 3.3.2 Evaluate use cases for precise care eXams

PHM of precise eXam intercepts feature omics and health determinant data in HPO Gen AI to educate medicine on personalised BM (Sbitan et al., 2025). An equitable, genome, and innovative approach screen early disease to target treatments for PHM efficiency (Ory et al., 2025). Evaluating genomic metrics ensures better accuracy and reliability of intercepts in clinical applications (Roberts et al., 2017; Whirl-Carrillo et al., 2021). Table 5 provides an overview of the metrics used to assess classifiers, with XAI an evaluation of HPO in PHM.

**Table 5:** Metrics for precise care eXams from pre-eXams.

Category	Specific metrics	Outcome/description
Implementation and access	Accessibility to personalised care	Proportion of the populace accessing precise eXams
	Cost reductions in healthcare	Effective targeting of precise eXams reduces events
Predictors and intercepts	The efficiency of an intervention	Measuring the impact of early biopharma or nutrient eXams
	Target therapy success rates	Statistics on the efficacy of a precise eXam
Clinical and personal efficacy	Treatment response rates	Percentage of positive outcome to a precise eXam
	Improve the quality of life	Measure of HPO to assure wellbeing through intercepts

A PHM GPT-5 landscape for rapid BM deployment of biomarker-driven predictive pre-eXams for citizens is a fair choice, with quantum-inspired algorithms in biopharma consortia poised to transform precise eXam intercepts (Flöther et al., 2025). HPO algorithms that rapidly analyse complex data records and images within multi-dimensional community spaces enable the discovery and assignment of novel biomarkers and drug predictive responses, necessitating enhanced evaluation metrics for classifications (Flöther, 2023). Quantum biopharma accelerates drug discovery, optimises clinical trial design, and refines personalised interventions, in a landscape of precise medicine with a need to steward reform or bias by continuous monitoring (Jeyaraman et al., 2024).

#### 3.3.3 Fairness through bias mitigation in AI-driven classifications

Fairness through bias mitigations is multicomplex in BM, while equitable access to genomic predictive health pre-eXams and precise care eXams is a national health right in a standard digital age for citizen choice to personalise their journey (George, 2025). Substantiating metrics are fair is a moral imperative, with techniques such as reweighing or adversarial debiasing an indispensable part of AI-driven classifications in diverse populations (Rabonato and Berton, 2024). The author explores bias mitigation extensively in a paper titled “Fit Lifecycles in Analytics” (Henry, 2025). Table 6 shows the key bias mitigation metrics to evaluate PHM fairness in HPO lifecycles (Genomics England, 2021; Our Future Health and NHS, 2022).

**Table 6:** Bias mitigation in the real world for the PHM of HPO.

Metric	Description	Instance of application
Fairness ratio (FR)	Ratio of positive outcomes of privileged vs. underserved groups	Bias in polygenic risk scores for chronic disease risks with FR
Accuracy in subgroup	Classifier accuracy in demographic subgroups on disparity	Accuracy improves in underserved groups during screens post-mitigation
Statistical parity	Assess similar outcomes across groups in classification	Improvement in major condition risk stratification for post-bias mitigation
Disparate impact	Measure the disproportionate effect on the groups	Disparate impact improvements in fairness audit with AI HPO screen tools

## 4 Action human phenotype ontology transformation

Figure 2 illustrates international agreements that action HPO transformation, while Figure 3 highlights BM value-based care classifications through Cloud Regions. PHM agile groups that action transformation develop IT-Hybrid-ISO norms for BM adoptions with HIMSS maturity, ISO JTC1 SC 42 AI, ISO 15189:2022 and point of need conformance across UK ICBs that standardise HPO practice (ISO, 2022; Healthcare Information and Management Systems Society, n.d.; International Organization for Standardisation, 2017; The Association of Clinical Biochemistry and Laboratory Medicine, 2023; UK Parliament, 2022). As nations differ in their choice of ecosystem actions, consider harmony from Section 4.1 Local Agile Groups for PHM, Section 4.2 Regional Higher Expert Medical Science Safety Agreements, Section 4.3 National HPO Value-Based Care, Section 4.4 Global Populace Health Management General Intelligence, and Section 4.5 Real-world AI vs. Clinical Practice Comparatives.

### 4.1 Local agile groups for PHM

Figures 2, 3 illustrate how agile group projects within local communities are the foundation for developing PHM ecosystems through agreements that streamline workflows. These initiatives ensure the safe implementation of HPO primary care in a cycle of predictors and intercepts tailored to local needs in neighbourhood centres (Victoriavaughan, 2024). The aim is to enhance wellbeing through data-driven engagement with NHS organisations and authorities while including public groups on valid scientific data themes that verify value-based outcomes (GOV.UK, 2022). Clinics adopt DNA pre-eXam risk stratification for disease segmentation, where pangenome assessments address HPO intercepts within the community (National Human Genome Research Institute, 2023).

In Figure 1, agile group development drives local PHM reform by establishing citizen workstreams with precise genome pre-eXams and eXam intercepts at local need points. These AI-driven health initiatives identify determinants of disease while aligning genomic nature for unique needs as socio-environmental factors nurture transformations in patient care and public health (Salgado et al., 2020; Vasta, 2023). Locally, HPO agile groups cooperatively and securely platform future health using structured knowledge and equitable tools (NHS England, 2024a; Whirl-Carrillo et al., 2021). Thus, the PHM of data resources for local HPO access is a progressive, agile AI project for BM evaluation and agreement on what is classified (NHS England, 2021b; The AI Safety Institute, 2024).

### 4.2 Regional higher expert medical science safety agreements

Figures 2, 3 showcase the PHM programme across UK ICB regions and international Cloud regional centres for Higher Expert Medical Science Safety (HEMSS) in HPO agreements as a primary care. Public health and patient safety transcend world regions, while a proposal for HEMSS steers the HIMSS assents for HPO maturity (Burrell, 2023; Gargano et al., 2023). HEMSS for public inclusiveness and stakeholder engagement across diagnostics elevates classifiers in safe spaces (GOV.UK, 2023b). To explain regional HEMSS as a steward, consider the following:

1. Higher infrastructure advances AI BM statistical-based predictive analytics for precise care with secure-scalable data management for real-time genome health as regional hub centres’ upstream factors for cluster insight on HPO primary care (Kitsios et al., 2023).
2. Expert agile algorithm development generates insights via factors in PHM classifications (Ahmad and Wasim, 2023). Agile stewards train data and apply XAI, leading to best HPO practices in regional programmes as PHM benchmarks support AI-BM audits (Ema et al., 2023).
3. Medical practitioners and pharmacists evolve the best-in-regions to enhance ecosystem decisions on data for best biopharma practice (BMJ Group, 2023). Science stewards’ ethical predictors and BM intercepts in HPO primary care to mitigate bias (Sahan et al., 2024).
4. Science in phenomics and social factors integrate HPO themes that bridge micro–macro factors in which a PHM digital voice develops BM (Frisinger and Papachristou, 2024). HPO transformation is a standard of future healthcare with valid classifications for adoption (Hameed et al., 2024).
5. Safe public health and patient welfare stewards conceptualise the enormity of the BM risks from HPO-structured knowledge (Endalamaw et al., 2024). Global digital health aligns regions with benchmark classifications as ecosystem PHM safety engages nations on predictive health pre-eXams and eXam intercept adoptions (Digital Health, 2023).

### 4.3 National HPO value-based care

Figure 3 illustrates national HPO value-based care while the manuscript contrasts transatlantic ecosystem reforms with surveys and assessments that address stakeholder variation to reduce infant mortality and enhance life expectancy: In 2021, the US infant mortality rate was 5.4 deaths per 1,000 live births, compared to the UK’s lower rate of 3.7 (CDC, 2024; Office for National Statistics, 2023). The UK’s average life expectancy of 78.6 years exceeds the US at 77.5 years (ONS, 2024; Centers for Disease Control and Prevention, 2023). National genomic screens for predictors and intercepts mitigate infant mortality and increase life expectancy, while DNA passport data exchanges harness citizen choice for HPO classification opt-in (Mayeur et al., 2023).

Figure 2 further showcases the UK and US Memorandum of Understanding on AI-HPO frontiers and advances science data themes with standard and tools agreements (International Organization for Standardisation, 2018). National UK ICS assessments and US CMS surveys for BM value with AI design propel HPO semantics and monitoring to enhance support decisions through classifications (de Mello et al., 2022; Jing et al., 2023). The UK GMS reform for predictors and intercepts in rare and major diseases is a “Generation study” and informs on “Our Future Health” (NHS England, 2024b; Our Future Health, 2021). NHS AI Ops align genome external quality assurance with BM performance monitoring in neighbourhood clinics with phenome value increasing through biobank access to data over time (UK NEQAS, 2023).

Figure 1 depicts AI-HPO transformation in classifiers to improve comprehension of mental and physical conditions, like any ecosystem that strives for value-based primary care (A. S. for P. Affairs (ASPA), 2024). US Institutes, like the Johns Hopkins School of Medicine-Population Health Office, plan future metrics in HPO management (The American Journal of Managed Care, 2023). Collaborating on BM evidence from research advantages Federal Centres in PHM value-based care (Lewis et al., 2024). A CMS-integrated care resource for digital HPO across the US would benefit from UK data and insight into their ICS (CMS.GOV, 2023a). Making HPO primary for all of us sets standard operations for equitable, efficient, and accredited organisation with valid PHM surveys (CMS.GOV, 2023b).

### 4.4 Global populace health management general intelligence

**Figure 4:**
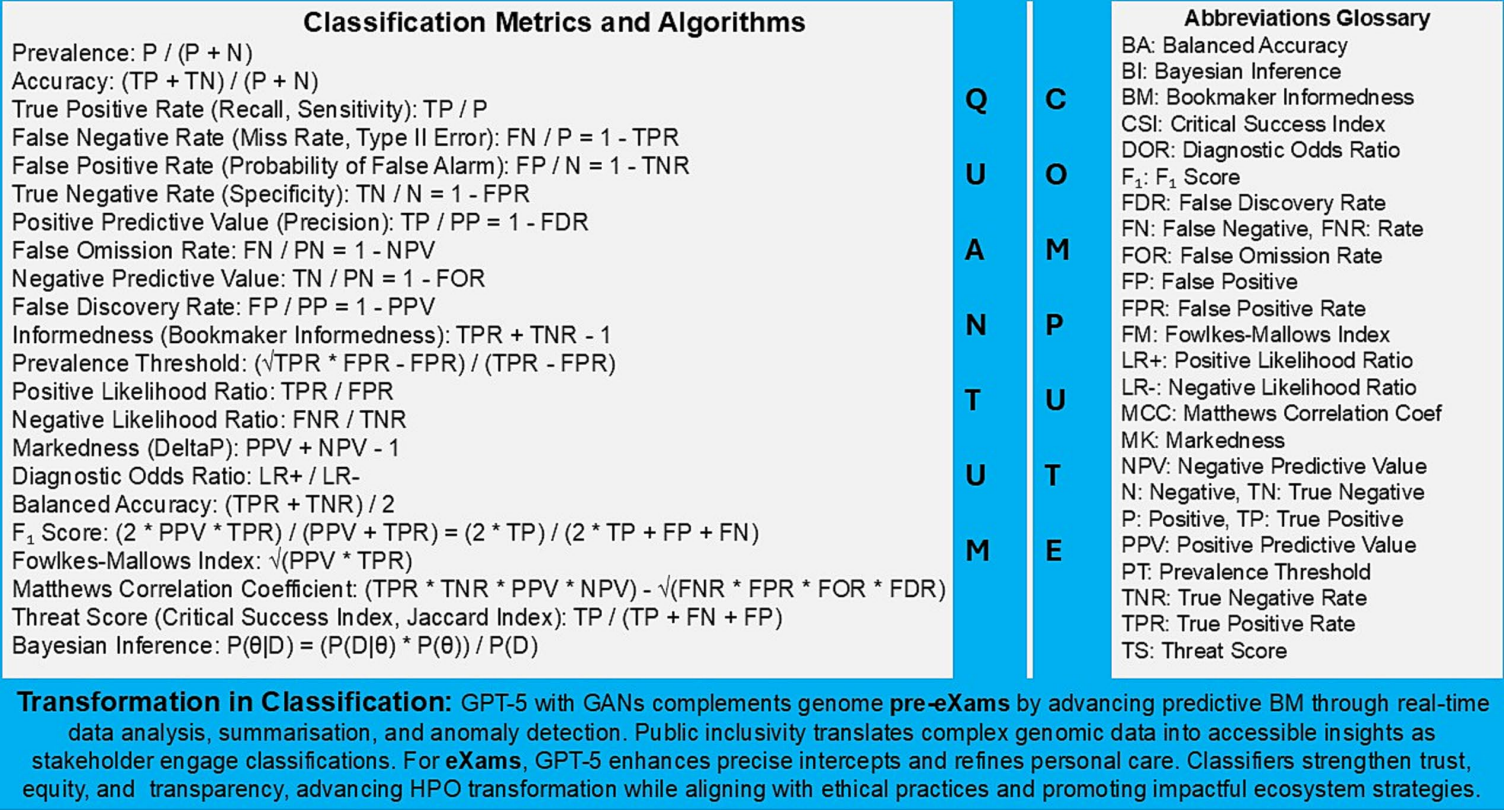
Population health management, the HPO classification transformation through evidence based metrics.

Figures 3, 4 showcase global transformation as we define the PHM of HPO with expert agile groups of individuals distributing general intelligence that features outcomes within the group through metrics (Kindig and Stoddart, 2003). World general intelligence expands as national institutions align physical wellbeing with factors in health promotion, disease prevention, and care intercepts, while informatics also enables parity (Kindig and Stoddart, 2003). A review set apprehensions on General Purpose AI use with debate on the frontier, advanced, and specialised algorithms available (GOV.UK, 2024b), which would align the classifiers and metrics shown in Figures 3, 4.

The global PHM of each HPO in an ecosystem with informatic handling and data protection can predict trends and identify risk groups with optimal stratification that segment disease and target intervention (HIPAA Journal, 2019; GOV.UK, 2023c). PHM action Trusted Research Environments with data alliances such as multi-omics, physiology, images, social determinants, and geographic information, which align with ethics and guardrails (UK Research and Innovation, 2023b; UK Health Data Research Alliance, 2024). Public awareness from urban concepts to neighbourhood centres emphasises that an ecosystem improves outcomes through general intelligence, like the factors that impact mental wellbeing (Duncan et al., 2021).

General intelligence unveils what we as communities consider successful HPO projects in PHM, including newborn genome rare disease to align infant gene editing, mental health disorders in adolescents for early intercepts, and adult CVD polygenic risk scores with imaging for personalised learning (Genomics England, 2021; NHS England, 2019; Caleyachetty et al., 2021). Lifetime access to HPO primary care delves deep into micro-omics and macro-environments to reduce hospital admissions, medication adversity, and social service demands as BM transforms to address comorbidity, polypharmacy, and pharmacogenomics challenges in a proactive wellness culture (World Health Organization, 2023b; Abel et al., 2018; HMC Architects, 2022).

General intelligence is at the heart of the matter with artificial vision and languages for HPO primary care that review risk scores to best inform on risk, such as new-onset atrial fibrillation by analysing polygenic scores and images with patient demographics, medical history, and lifestyle factors supported by statistics (Crea, 2023; Olesen et al., 2012). Ecosystem-safe space best instances HPO-AF as digital twins through public/private partnerships for personalised therapeutics, as science and technology assure intercept accuracy to reduce lifetime complications as a public health initiative (Fischer et al., 2024).

### 4.5 Real-world AI vs. clinical practice comparatives for classifications

In Figure 3, real-world AI evidence vs. clinical practice informs that science and technology can deliver value-based care in predictors and intercepts. Figure 4 presents metrics that support classifications, while Tables 7, 8 instance real-world use cases for predictive health pre-eXam and precise care eXams.

**Table 7:** Real-world AI in clinical practice comparatives for pre-eXams.

Traditional practice metrics	Genomics AI-driven metrics	Statistical comparison
Genetic queries not affirming HPO (missing data)	Increased discovery through AI-driven DNA imputations for pre-eXams	A 1.8-fold increase in GWAS sample size, for loci discovery (An et al., 2023)
Evaluation with conventional infant ICU management	Metrics aligned with GWS to diagnose >200 rare diseases for management	46% of patients assigned HPO rare disease from a pre-eXam (Amin et al., 2024)
Risk Assessment without PRS limit HPO efficacy	Align multi-ancestry (MA) AI analysis improves PRS pre-eXam	MA PRS such as T2D explain X% of familial risk with AI (Guo et al., 2025)
Family history for cancer risk misses’ oncology carriers	AI-Enhanced Genome Risk, PHM screening pre-eXam	CSGs have a prognosis of up to 60% HPO oncology (Swisher et al., 2025)

CDs comorbid diseases, CSG cancer susceptibility genes.

**Table 8:** Real-world AI for clinical practice digital twin eXams.

Traditional medic, research, and biopharma metric	AI-driven HPO metric	Expected comparative for acceptance
SC-VOC crises (VOCs) and hospitalisation episodes	CRISPR-Cas9 gene editing to HBF and eliminate VOCs	97% of patients were free from severe VOCs > 12 months
Lengthy and biased clinical trial person selection process	AI-powered digital twin eXam for rapid, diverse trial recruitment	Time reduced for eligible persons cost savings; and diversity
Low patient retention, trust and satisfaction in clinical trials	AI-powered digital twin modelling eXam to improve patient engagement	Increase in patient retention rates and high patient satisfaction
Delayed-inaccurate detection of ADRs seeks PRS	Gen AI eXam for real-time ADR surveillance and prevention	Reduction in adverse events during clinical trials.
Variable-unpredictable drug response in cancer treatment	Multi-omics precise eXams that personalise treatment	Improvement in overall HPO oncology health outcomes

SC-VOC sickle cell vaso-occlusive crisis, ADR adverse drug reaction, PRS polygenic risk score.

Table 7 highlights the transformative HPO from genomics predictive BM pre-eXams, demonstrating how AI-driven solutions enhance outcomes across primary care as a digital twin predicts disease (Alam et al., 2024). Table 8 shows AI-driven HPO applications with digital twin biopharma eXams, with row 1 demonstrating a gene therapy eXam (Anand et al., 2024). Table 8, rows 2–5, contrasts traditional and AI-enabled metrics for HPO optimisation, patient engagement, adverse drug reaction surveillance, and drug response prediction.

Figure 4 and Table 8 source digital twin metrics with a note to the reader that 90% of healthcare spending is on chronic disease while accurate HPO Gen AI (eXams) intercepts built from real-world data will drive efficiencies through safer outcomes (Criteriumadmin, 2024; MedTech Intelligence, 2024; Vidovszky et al., 2024).

- Row 2 Digital twin recruitment enables precise HPO targets in populations, improving enrolment rates and diversity in patient recruitment metrics. Operational metrics, such as low pre-eXam screen failures and faster recruitment times, reflect enhanced efficiency (Criteriumadmin, 2024; Kasahara et al., 2024).
- Row 3 Digital twin engagement on personalised experiences boosts patient satisfaction and retention in citizen-centric approaches for PHM. Improved retention indirectly enhances recruitment by reducing the need for replacements in patient recruitment metrics (Criteriumadmin, 2024; Terceiro et al., 2025).
- Row 4 Adverse drug reaction detection with AI provides real-time monitoring, improving safety reporting rates and data accuracy, while metrics detect and prevent events in BM updates (Criteriumadmin, 2024; Qiu et al., 2024).
- Row 5 Drug response with multi-omics data integration and AI-driven analysis provides BM objective predictions of treatment response, enhancing data quality and enabling the PHM of HPO intercepts eXams in data quality metrics and objective assessment (Criteriumadmin, 2024; Robert and Gautheret, 2022).

From Tables 7, 8, advanced AI represents an HPO transformation to best understand, learn, and apply knowledge through metrics, moving beyond the task-specific research improvements to real-world implementation of classifications. Quality multimodal data for analytics feature omics, images, and socio-environmental factors across records, while real-world digital twins evidence disease predictors (An et al., 2023; Amin et al., 2024; Guo et al., 2025; Swisher et al., 2025; Alam et al., 2024) for intercepts (Kasahara et al., 2024; Terceiro et al., 2025; Qiu et al., 2024; Robert and Gautheret, 2022) as PHM metrics align HPO classifiers (Criteriumadmin, 2024). Stewarding benchmark classifiers in primary care agreements is the new frontier of PHM, as depicted in Figures 1–3.

## 5 Global HPO transformation discussion

This section discusses the transformative potential of Federated Learning and Quantum Computing combined with GPT-5 to securely train hybrid ML models on sensitive genomic datasets without exposing raw data (Deshprabhu, 2023; Dhade and Shirke, 2024). This HPO transformation standardises real-world applications, including newborn generation studies for predicting rare diseases and normalising polygenic risk scores for non-communicable disease prevention. It also optimises multi-omics for drug selection and targeted therapies (Genomics England, 2021; Our Future Health and NHS, 2022; Biobank, 2025; Pharmaphorum, 2025).

**Figure 5:**
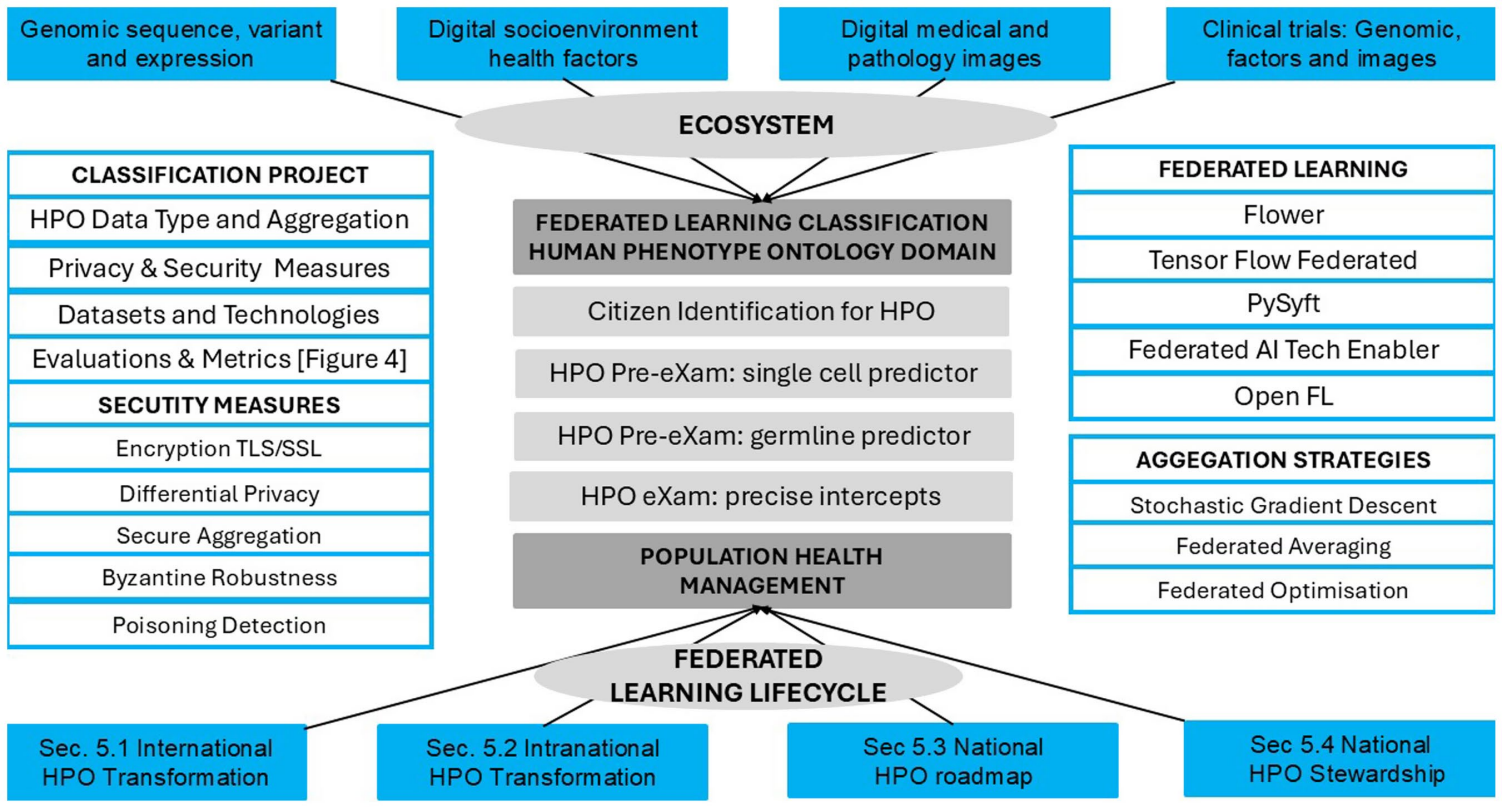
Population health management, the HPO transformation with privacy in federated learning.

Figures 4, 5, along with Tables 9, 10, highlight standardised PHM metrics for HPO transformations that federate BM learning within secure environments. GPT-5 classifications connect personalised applications with international, intranational, and national cooperation. Subsections 5.1–5.4 examine global approaches to public inclusiveness and stakeholder engagement, scaling classifiers through Higher Expert Medical Science Safety (HEMSS), a steward for the PHM of HPO.

**Table 9:** International key expert areas guidance on HPO transformation.

Key expert areas	Global	United Kingdom	United States
PHM strategy and HPO transformation	WHO Frameworks and UN SDGs 3,8,17	Health & Care Act 2022 NHS 10-year structure	Affordable Care Act 2010 National AI Initiative
AI safety and gen AI explainability	Global AI partnership and UNESCO	AI Safety Institute Evaluation for Research Resource Clusters towards XAI and regulation of PHM for HPO	
Algorithmic bias and validation	Global ISO/IEC TR 24027:2001 (Bias)	Alan Turing Institute on AI Bias mitigation	NIST risk manage bias to validate healthcare
Pre-eXam genomics classifiers—quantum	G4HEALTH, HPO Portals	Genomics England studies and programmes	NIH All of US programme with partnerships
Life sciences/ pharma eXam classifier	Global Biobanks and Consortia	Life Sciences Bio Pharma Consortia	
ISO standards and HIMSS	ISO: 15189:2022, ISO IEC JTC1 SC 42, ISO/IEC 27001 (see below) Higher Information and Management System Society maturity		
Security risks – quantum	Information Security Management System	Digital Regulation Service: Cybersecurity	NIST Cybersecurity to manage risk
Privacy: federated learning	GDPR for global data privacy	GDPR Consent and DUAB	HIPAA (Amendments)
Workforce training and upskilling	Global Medical Associations	Digital Skills Partnerships	National AI Initiative Office

**Table 10:** HPO transformation roadmap.

Key aims	Core activities	Expected outcomes	Evaluations metrics
Phase 1 (HIMSS mature development) – HPO foundation and data integration			
Establish data infrastructure and define key HPO objectives.	Data storage and standardisation; data governance policies; identification of HPO datasets; stakeholder mapping.	Integrated data platform; defined HPO goals and target populations baseline health metrics; initial stakeholder alignment on PHM	Data accuracy; stakeholder participation; clarity of HPO objectives; number of data sources integrated.
Phase 2 (HIMSS/HEMMS)—Pre-eXam biological models and predictive analytics			
Develop predictive models and assess intervention impacts.	Multi-scale BM development; Machine Learning for risk stratification; simulation of Pre-eXam predictions; validation of outputs.	Predictive BM for disease segmentation; identify high-risk populations; prioritised interventions; quantified impacts; roll out the pre-eXam concept across policies	BM accuracy; stratification power; reduction in disease burden—simulated; intervention cost-effectiveness or pre-eXams (estimated)
Phase 3 (HIMSS/HEMSS)—the eXam implementation and intervention			
Deploy targeted interventions and monitor health outcomes.	Evidence-based intervention implementation; real-time health monitoring; adaptive strategies based on feedback; stakeholder engagement.	Improved health outcomes; real-world validation of models; optimised delivery of interventions; enhanced satisfaction from public and science and technology stakeholders	Real-world health outcome changes; adherence to interventions; cost-effectiveness of real-world interventions; stakeholder satisfaction levels.
Phase 4 (HEMSS principal adoption) – classify, optimise and sustain			
Scale classifications integrate into choice of routine practices and ensure sustainability.	Sustainable implementation model development; HPO integration into an ecosystem; continuous improvement; public engagement and education initiatives.	Institutionalised HPO practices; long-term health improvements; increased public awareness with DNA inclusivity and engagement; secured sustainable funding; socioeconomic success.	Long-term health trends; sustainability of classifications with stakeholder engagement on metrics; cost savings and resource efficiency.

### 5.1 International HPO transformation

In Table 9, international organisations influence national ecosystems to advance HPO transformation through PHM cooperation for Biological Models (BM). Standards such as ISO 15189:2022 and IEC JTC 1/SC 42 guide ecosystem AI advancements at points of care to meet HPO needs (Royal College of Pathologists, 2022; International Organization for Standardisation, 2017; The Association of Clinical Biochemistry and Laboratory Medicine, 2023). Global HIMSS maturity is advisory and voluntary support for BM development and AI laboratories, while HEMSS principles, as proposed by the author, act as stewards for PHM adoption of classifications (HIMSS, 2018; HIMSS, 2024).

From Table 9, global harmonisation of HPO practices for predictive health genomics in pre-eXams and precise care eXams emphasises the importance of maintaining ethical and equitable standards. As illustrated in Table 9, nations and citizens establish their classifier agreements, paving the way for a future shaped by advancements in quantum intelligence, federated learning, and GPT-5 across key expert domains for stewardship of classifiers.

Figure 1 and Table 9 depict the aim for the PHM of genomics, highlighting how national quantum computing enhances HPO classifiers. AI safety evaluations under a UK-US Memorandum of Understanding safeguard sovereignty and privacy while expanding analytical capacities for HPO (GOV.UK, 2023a; Tech UK, 2024). Quantum intelligence accelerates genomic research, enabling rapid and precise medical applications to inform public health policies (Flöther et al., 2025; Flöther, 2023; Jeyaraman et al., 2024).

Figure 2 and Table 9 illustrate assessments of federated learning empowering nations to integrate genomic data into ecosystems ethically. A decentralised AI training approach addresses transparency and informed consent challenges while safeguarding sensitive data. Federated learning reinforces privacy and ensures fair genomic analysis across populations, supporting PHM progress and advancing public health (Deshprabhu, 2023; Dhade and Shirke, 2024; Tech UK, 2024).

Figure 3 and Table 9 emphasise the role of Gen AI, such as GPT-5, in overcoming disparities across organisational sectors. BM-based Gen AI enables cost-effective, value-based care classifications through tools and synthetic data learning, while advancements are fair and equitable, ensuring sustainable PHM with widespread access to HPO benefits (Quazi et al., 2024a; OpenAI, n.d.; Berman, 2024).

### 5.2 Intranational HPO transformation for just classifications

The integration of high-throughput genomics with quantum intelligence represents a paradigm shift, simultaneously enabling the PHM of HPO. ML, clustering, evaluation, and stewarding identify disease-associated genetic variants, discovering biomarkers, and optimising drug targets (UK Research and Innovation, 2023a; GOV.UK, 2023a; NHS, 2025). This section discusses national healthcare system variances for HPO transformation in Section 5.2.1, which impacts intranational opportunities and challenges through classifiers in Section 5.2.2.

#### 5.2.1 Intranational variances for HPO transformation

This section is a comparative analysis that debates national healthcare systems, focussing on the intranational impact of their structures, policies, and technological adoption for implementing genomic predictive pre-eXams and eXam intercepts globally.

The UK Health and Care Act 2022 aims to structure PHM and stratify HPO risk for disease segmentation in centres (NHS Providers, 2022; Wale et al., 2024). HEMSS stewards’ adoption of genome services in germline or cell WGS pre-eXams (Hayward et al., 2023). Biopharma consortiums that develop precise care eXam intercepts require oversight in a HEMSS stewardship proposal (UK Research and Innovation, 2023a; GOV.UK, 2023a; NHS, 2025).

The EU faces challenges due to its member states’ heterogeneity, such as Finland’s progress with active DNA donation programmes in contrast to slower engagement (FINNGEN, n.d.; Ireland, Health, and Safety Executive, n.d.). The EU AI office and pharmacogenomics projects highlight the challenges of system harmonisation and the need for global stewardship (European Commission, 2024; Blagec et al., 2018).

With its state and private health systems, the US presents a complex PHM as national insurance aims for HPO value-based care (Rovner, 2024; CMS.GOV, 2024). The Executive Order to advance technology signals an attempt to address challenges through robust stewardship and HPO-AI policies on federal, state, and local developments (White House, 2023).

Australia’s genomic integration success is achieved through a centralised health reform system that aligns with sound principles and serves as a compelling contrast to the EU and the US, demonstrating the potential of a unified approach to streamline HPO transformation and improve public health outcomes (Australian Government, 2020).

China’s centralised healthcare system, supported by “Healthy China 2030,” employs quantum intelligence and high-throughput genomics to advance the potential for classifications, demonstrating global competitive potential while aligning with international health promotion efforts (World Health Organization, 2016).

From Table 9, global harmonisation of HPO practices for predictive health genomics in pre-eXams and precise care eXams emphasises the importance of ethical and equitable standards. As illustrated in Table 9, classification is pertinent to all national structures, while providing for their unique priorities and levels of stewardship.

#### 5.2.2 Debates on classification opportunities and challenges

National ecosystems debate classification opportunities and challenges associated with the global implementation of AI-driven agreements, technology, and metrics for health and wellbeing as stakeholders engage in economic growth, as depicted in Figures 1–4 (UNEP-UN Environment Programme, 2021; EMBL, n.d.). PHM opportunities from standards were assessed in Section 3, while PHM challenges were addressed in HPO transformation in Section 4, culminating in a culture that will debate agile genomic pre-eXams for seamless eXam intercepts (Magistretti and Trabucchi, 2024).

Arguments for AI-driven classifiers in intranational pre-eXams and eXams intercepts:

1. Predictive AI-driven pre-eXams utilise expansive genomic and phenomics datasets to identify individual disease predispositions accurately. This enables early intervention strategies and facilitates personalised preventive care (Bagger et al., 2024).
2. The rapid analysis of complex multi-omics data enhances diagnostic efficiency in AI-driven drug and dose eXams, providing faster and more precise diagnoses that establish new benchmarks in personalised medicine (Carini and Seyhan, 2024).
3. Standardised interpretation of genomic data within AI-driven classifications reduces clinical variability worldwide, ensuring equitable healthcare delivery across diverse settings with notable economic benefits (Dorocka, 2024).
4. Global harmonisation of pre-eXams and eXams promotes responsible, universally guided processes that reform citizen outcomes within interconnected bioinformatic ecosystems (Alowais et al., 2023).

Arguments against global pre-eXams and eXams:

1. Integrating AI-driven classifications with traditional diagnostics and conventional medical practices poses significant complexity and demands extensive adaptation and investment, as illustrated and simplified in Figure 1.
2. Disparities in data formats and standards across national healthcare systems create substantial challenges in achieving data standardisation, as shown and simplified through multiple national agreements in Figure 2.
3. The global implementation of AI-driven pre-eXams and eXams requires extensive computational infrastructure and resources, which are not evenly distributed among nations, as partially illustrated for progression in Figure 3.
4. Applying pre-eXam and eXam concepts may complicate medical processes with an unnecessary validation, introducing implicit and explicit bias risks. Yet, HEMSS metrics and federated classifiers address debates in Figures 4, 5.

### 5.3 National HPO roadmap inclusiveness and engagement

A national roadmap for the PHM of HPO which is inclusive for public health and patient safety is as dependent on citizen-informed consent as on stakeholder engagement in quantum computing. Section 5.3.1 discusses the UK government milestones for HPO transformation in phases. Section 5.3.2 debates that informed consent and quantum intelligence are key resources to realise the PHM map.

#### 5.3.1 National HPO transformation roadmap

The UK Government’s NHS milestones (G. D. Service, 2024) outline a national strategy, an action plan, and an adoptive mission (UK Government, 2021; GOV.UK, 2025c). These milestones emphasise the necessity of public inclusiveness in genomic pre-eXams and eXams through multiple stakeholder engagements and agreements, enabling transformative advancements in HPO primary care, as detailed in Table 10.

Table 10 shows that phases 1–4 require robust technologies and oversight. A national roadmap for phases 1–3 federates learning, quantum computing, and GPT-5, providing HPO transformation, as illustrated in Figures 4, 5. Public inclusiveness and comprehensive communication ensure that HPO transformation remains accessible and balanced. This genomics phase approach to stakeholder engagement moves from collaborative to cooperative HPO to support public health and patient safety with agreed classifications depicted in Figures 1, 2, as explained in Phase 4.

- Phase 1 of the HPO foundation and data integration uses federated learning predictors and intercept classifiers that sustain privacy (Figure 5). Federated learning addresses challenges in integrating distributed datasets while safeguarding citizen rights, as demonstrated by systematic reviews on privacy-preserving techniques in healthcare data sharing (Xu et al., 2020). These methods are foundational for ethical HPO and provide the groundwork for achieving the transformation required for broad PHM adoption and sustained success through classifiers (Gargano et al., 2023).
- Phase 2, Pre-eXam BM predictive analytics, deploys quantum intelligence for national WGS and HPO pre-eXam classifications with health determinant metrics, which enhances risk stratification through Generative AI, such as GANs with pangenomes (Figure 4). Quantum intelligence in this phase strengthens computational capabilities for complex BM development, exploring predictive models in personalised medicine (Oluwafemidiakhoa, 2024). Classifications also use quantum cybersecurity, which is vital for the future of BM predictive analytics (Nguyen et al., 2024).
- Phase 3 focusses on lifetime eXam implementation and intervention, incorporating the personalised GPT-5 use case to facilitate disease segmentation intercepts (Figure 4). GPT-5 aligns ongoing research on the ethical and practical implications of large language models in healthcare, particularly their role in clinical decision support for intercepting HPO (Adarkwah et al., 2025). Experts and national stewards should explore the impact of GPT-5 deployment to maintain a robust ecosystem that addresses public health needs and enhances patient safety (Quazi et al., 2024b).
- Phase 4 focusses on HPO classification and optimisation, with an increased PHM capacity for BM capabilities. Governance ensures PHM transformation by highlighting AI’s critical role in advancing ethical considerations, data security, and equitable access (Mittelstadt, 2019). These advancements promote evidence-based practices as the HPO roadmap achieves intranational wellbeing goals that align seamlessly with intranational targets and sound stewardship that unites nations for good health and wellbeing (United Nations, 2023).

#### 5.3.2 Primary HPO resources are informed consent and quantum intelligence

The roadmap for national HPO transformation identifies public inclusivity with informed consent and stakeholder engagement on quantum intelligence as the key foundational elements. These components underpin an action plan, demonstrating that the financial investment required can be justified, as discussed in Section 3.1.1 (GOV.UK, 2021; NHS, 2023; NHS Health Education England, 2020; NHSE England, n.d.). National PHM supports research cluster evaluations in genomics phases, integrating NHS plans in AI infrastructure for HPO transformation (G. D. Service, 2024; UK Government, 2021; GOV.UK, 2025c; Department of Science, Innovation and Technology, 2023; GOV.UK, 2025b; Biobank, 2025; Pharmaphorum, 2025; UK Research and Innovation, 2023a; GOV.UK, 2023a; NHS, 2025).

Public inclusivity debates informed consent in cluster and decentralised trials, which must sustain public trust and ethical transparency in genomic classifiers (Arellano et al., 2023). While data save lives, citizens must be well-informed about how their genomics data will be used, analysed, and shared (NHS England, 2023). By respecting the citizen’s genomic autonomy, individuals can make informed decisions about BM participation while interdisciplinary dialogue guides a DNA opt-in (Tribe, 2022; Alvarellos et al., 2023). Inclusive genomics protocols ensure equitable public access to good health as policymakers engage stakeholders on HPO transformation (Panteli et al., 2025).

Stakeholder engagement with quantum intelligence and transformative capabilities for large-scale genomic and social factor datasets optimises the PHM of HPO outcomes through enhanced computational power (Singh and Raj, 2025). Quantum computing advances PHM goals such as citizen risk stratification, disease segmentation, and targeted interventions, aligning genomic phases to improve practices (Dixon et al., 2024). However, the challenges of transparency, bias, and accountability in quantum computing necessitate stewardship in AI ethics and the responsibility to sustain public trust among stakeholders via national classifications (Bano, 2018).

HEMSS builds inclusivity and engagement principles for HPO policy with roadmap actions and an ethical bridge, linking standardised approaches shown in Tables 1–3. One further connects HPO digital twin metrics in Tables 4–8 with international guidance detailed in Table 9 for responsible PHM in Table 10 with public health quantum intelligence. While DNA-derived data hold immense potential, stewardship is essential to prevent misuse while delivering value-based care in a federated quantum public health ecosystem, balancing equitable PHM with HPO transformation, as illustrated in Figures 1–5.

### 5.4 National PHM balance through classifications and stewardship

This discussion explores the role of classifications and stewardship in achieving a balanced national approach to Population Health Management (PHM). The manuscript highlights the major advantages and minor disadvantages of classification stewardship, focussing on its impact on individual wellbeing and socioeconomic success. Building on the analysis in Sections 5.1–5.3, the author extends the PHM transformation debate and re-evaluates the manuscript tables to illustrate key metric points related to Human Phenotype Ontology (HPO).

National PHM is underpinned by advancements in federated learning (Deshprabhu, 2023; Dhade and Shirke, 2024), quantum intelligence (Flöther et al., 2025; Flöther, 2023; Jeyaraman et al., 2024), and GPT-5 (Adarkwah et al., 2025; Quazi et al., 2024b), which collectively enhance classification accuracy in the dawn of digital stewardship. Together, these advancements provide a comprehensive ecosystem for understanding how classifications and stewardship relays an equitable, efficient, and transparent PHM ecosystem. Sections 5.4.1 and 5.4.2 present a vision for PHM transformation through BM pre-eXams and eXams, highlighting their transformative potential, while Section 5.4.3 informs on digital PHM ecosystem limitations in transformation to a HPO ecosystem.

#### 5.4.1 Genomic pre-eXam classification—advances in predictive health

The foundation of pre-eXams lies in whole-genome sequencing (WGS) assessments, which uncover genetic predispositions and provide actionable clinical insights (Amin et al., 2024). These assessments are enhanced using pangenomes, improving specificity and sensitivity (National Human Genome Research Institute, 2023). They are integrated into HPO policies for genomics advancements, including newborn screening (Genomics England, 2021), future health programmes for high-risk groups (Our Future Health and NHS, 2022), and multi-omics metrics outlined in Tables 5, 7. Table 10 outlines a roadmap for Phase 2 for developing valid and cost-effective classifications explainable through the Pre-e(X)am. The simplification of scientific reforms is illustrated through classification calculations in Figure 4, addressing the potential for expansive applications:

- Monogenic insights: AI-driven genomics detect single-gene mutations, enabling early diagnosis and intervention for rare disorders, as outlined in Table 7 metrics.
- Polygenic and oncology insights: Genomic AI platforms assess multi-gene risks and evaluate hereditary susceptibility to major conditions, refined in Table 7 metrics.
- Functional insights: FAVOR GTP annotations analyse gene–environment interactions, advancing HPO predictive BM through tools outlined in Table 5.
- Epigenetic insights: GENOME GTP links gene modifiers to stress-related disorders, representing epigenetic insights within BM of HPO using tools in Table 5.

From genome data to multi-omics blueprints, digital twin simulations in Tables 7, 8 refine pre-eXams by enhancing BM responses with greater precision. These simulations incorporate mathematical ecosystems, such as Bayesian inference for conditional probability analysis and multi-variable differential equations for dynamic BM in HPO, significantly advancing predictive health capabilities as shown in Figure 4.

#### 5.4.2 Biopharma eXams—translating predictions into precision care

Building on the predictive insights established through pre-eXams, biopharma eXams operationalise omics data into therapeutic applications, addressing barriers to innovation and equity to resolve real-world HPO-AI intercepts (Sbitan et al., 2025; Ory et al., 2025). While Digital Regulation Services may present challenges that slow innovation, effective stewardship can accelerate classifiers by incorporating valid bias mitigation and XAI methodologies, as demonstrated by metrics in Tables 6, 8, which are themselves driven by AI (Carini and Seyhan, 2024). Figure 4 illustrates key metrics that, while fit for purpose in eXams, may face rejection during national cost–benefit analyses of the proposed intercept. Table 10 outlines Phase 3 in a roadmap for developing classifications explainable through the e(X)am intercept in the HPO transformation.

The same four omics areas described in Section 5.4.1 drive advancements in biopharma eXams, with applications in the following areas:

- Monogenic eXams: Digital twins simulate therapeutic responses ranging from gene therapy to protein replacement therapies for rare conditions. Specific omics intercepts, supported by Gen AI as X, ensure transparency within a user-comprehensible ecosystem. Pre-eXam applications leverage eXam metrics detailed in Table 8, providing precise BM targets tailored to genomic interventions.
- Polygenic eXams: Pharmacogenomic and major condition assessments guide tailored interventions for complex genetic profiles. The interpretive of X ensures interventions remain comprehensible and accessible to diverse users. Fairness improvements, including subgroup accuracy gains, are supported by bias mitigation metrics in Table 6.
- Functional eXams: Biomarker discovery aligns multi-omics data with scalable solutions, integrating insights from exons to dark data for advanced engineering of oligonucleotide and protein therapies. X serves as the user-focussed interpretive framework, enhancing trial retention and satisfaction rates as detailed in the metrics from Table 8.
- Epigenetic eXams: Monitoring methylation supports personalised therapies, while real-time ADR surveillance through digital twin metrics in Table 8 minimises adverse events during trials. X provides a comprehensive ecosystem for interpreting epigenetic applications across diverse clinical contexts.

Biopharma eXams bridge the gap between genomic insights and therapeutic innovations through omics data with cost-effective digital twin simulations. These advancements optimise BM responses with remarkable precision in the PHM of HPO.

#### 5.4.3 The limitations of classifications with stewardship

Predictive pre-eXams and biopharma eXams offer transformative advancements for HPO, yet their reliance as lifetime predictors and intercepts introduces limitations. These challenges also emphasise the importance of ethical stewardship, public inclusiveness, and stakeholder engagement to establish a fair, equitable, and effective ecosystem. Below are five key limitations and the approaches to navigate them:

1. Overdiagnosis and overclassification: excessive reliance on classifiers can result in overdiagnosis and unnecessary interventions, which inflate healthcare costs and negatively impact patient wellbeing. Addressing this limitation requires structured stakeholder engagement, as outlined in Table 10 roadmap phases that balance prediction with precision, ensuring classifications remain grounded in meaningful and pragmatic applications.
2. Amplification of inequalities: AI models trained on non-representative datasets risks amplifying health disparities, leaving specific populations underserved or misrepresented by inaccurate predictors and intercepts. Bias mitigation strategies detailed in Table 6 incorporate diverse datasets and engage global biobanks, ensuring equitable and inclusive classifications. These approaches promote fairness in PHM and reduce systemic inequalities within HPO ecosystems.
3. Data privacy and security risks: the integration of large-scale genomic data introduces vulnerabilities to cyberattacks and unauthorised access. Federated learning for genomic information and quantum-safe cryptographic measures, as outlined in Table 5, are essential for protecting citizen informatics and genomic data integrity. These safeguards maintain trust in predictive healthcare systems while ensuring BM security within the PHM of HPO.
4. Algorithmic bias and fairness: bias within training datasets is a pervasive challenge that undermines classification reliability and ethical use. Statistical parity metrics and synthetic datasets, supported by Table 6, mitigate biases to enhance subgroup accuracy. These tools ensure that classifications are both justifiable and ethically sound, aligning outcomes with diverse clinical and population contexts.
5. Over-reliance on technology: dependence on AI risks diminishing the role of human-centred care and the vital patient-clinician relationship. As healthcare systems transition to HPO metrics, sustaining public health and patient safety requires augmenting practitioner roles rather than replacing them. Table 10 provides a roadmap to address challenges collectively, incorporating HEMSS to preserve value-based care and practitioner integrity.

Despite these minor limitations, pre-eXams and biopharma eXams continue to advance predictive health by using tools such as variant calling, functional annotation, and epigenetic profiling to drive personalised therapies. An HPO transformation strengthens public health by integrating it into a unified, data-driven ecosystem, presenting a major advantage for global health through the promotion of equity and sustainability across diverse populations. Moreover, classifications actively engage stakeholders through agile BM methods, emphasising public inclusiveness and being reinforced by stewardship in HEMSS to uphold value-based care.

## 6 Conclusion and policy implications

This manuscript has examined the transformative potential of integrating the Human Phenotype Ontology (HPO) into national Population Health Management (PHM) strategies, highlighting the crucial role of advanced digital infrastructure and AI. The preceding sections outlined the aims, assessments, classifications, metrics, and implementation necessary for successful HPO transformation. Discussions on the findings highlight the necessity of a unified, data-driven approach employing AI, including Federated Learning, Quantum Intelligence, GPT-5, and genomic insights, to reform personalised healthcare delivery.

An HPO policy could be collaboratively developed by the Department of Health and Social Care (DHSC) and the Department for Science, Innovation and Technology (DSIT). This policy would address public inclusivity, ethical considerations, regulatory frameworks, and infrastructural requirements to ensure the safe and effective deployment of PHM. A UK policy, based on a series of manuscripts by the author, could be adapted for other nations, such as the US, accounting for ecosystem heterogeneity to enable data governance, interoperability, and responsible AI usage in classifications for predictive health pre-eXams and precise care eXam intercepts.

Proposals such as Higher Expert Medical Science Safety (HEMSS) agreements introduce an AI Digital Regulation service stewardship model that provides a robust framework for ensuring patient safety and data integrity. This approach is adaptable to diverse national contexts while aligning with HIMSS and national infrastructures. Nations can draw on these insights to shape their HPO implementation strategies, ensuring compatibility with healthcare objectives and regulatory environments. A national HPO policy should be established in collaboration with Global AI Safety Institutes and AI Research Resources. These institutions and processes play a vital role in refining and supporting policies that align with international standards and ethical practices.

Collaborative roadmaps serve as guides for cooperation and facilitate knowledge exchange, promoting a harmonised approach to PHM within and across nations. The roadmap for HPO transformation within a UK policy proposition should follow a phased genomic approach: Phase 1 centres on data infrastructure and objectives; phase 2 develops predictive models; phase 3 focusses on targeted interventions; and phase 4 stewards’ classifiers. These interconnected phases provide a coherent framework for a national HPO policy, which could serve as a template for adoption by other nations. Such a policy must balance capacity with capability as quantum intelligence matures into these phases.

The global establishment of robust PHM evaluation and continuous improvement mechanisms is crucial to unlocking the potential of HPO transformation, resulting in improved patient outcomes and more equitable national healthcare ecosystems. Recommendations within this manuscript encourage dialogue among nations advancing healthcare innovation, paving the way for a future where personalised, data-driven public health and medicine become a practical reality. The final manuscript in this series will propose a UK HPO policy that is comprehensive yet adaptable, supporting classifications and stewardship to enhance public health, patient safety, and equity.

## Glossary

ACB: Association of Clinical Biochemistry
ADR: Adverse Drug Reaction
AF: Atrial Fibrillation
AI: Artificial Intelligence
AIDRS: AI Digital Regulation Service
AISI: AI Safety Institute
AMAM: Adoption Model for Analytics Maturity
ANNOVAR: Tool for functional annotation
API: Application Process Interface
BMA: British Medical Association
BMJ: British Medical Journal
BWA-MEM: Tool for data alignment
CCMM: Community of Care Maturity Model
CDs: Cardiovascular Diseases
CMS: Centers for Medicare & Medicaid Services
CRISPR-Cas9: Gene editing technology
CSG: Cancer Susceptibility Genes
CVD: Cardiovascular Disease
DL: Deep Learning
DNA: Deoxyribonucleic Acid
DNABERT: Tool for AI-Driven Analysis
DRAGEN: Tool for variant calling
DUAB: Digital User Access Bill
EHR: Electronic Health Record
EMRAM: E-Medical Record Adoption Model
FAVOR-GPT: Functional Annotation Variant Optimised Genomics GPT
FDP: Federated Data Platform
FR: Fairness Ratio
GA4GH: Global Alliance for Genomics and Health
GAN: Generative Adversarial Network
GATK: Genome Analysis Toolkit
GDPR: General Data Protection Regulation
GEN AI: Generative AI
Genome GPT: Genomic AI model for genetic analysis
GMS: Genomic Medical Service
GPT: Generative Pre-trained Transformer
GWAS: Genome-Wide Association Studies
HBF: Haemoglobin F
HEMSS: Higher Expert Medical Science Safety
HIE: Healthcare Informatic Exchange
HIMSS: Healthcare Information Management System Society
HIPAA: Health Insurance Portability and Accountability Act
HPO: Human Phenotype Ontology
HSCA: Health and Social Care Act
ICB: Integrated Care Board
ICS: Integrated Care System
IFRAM: Infrastructure as a Model
INDEL: Insertion/Deletion Mutation
ISO: International Organization for Standardization
IT: Information Technology
ITIL: Information Technology Infrastructure Library
LLM: Large Language Models
MA: Multi-Ancestry
Minimap: Tool for data alignment
ML: Machine Learning
NAS: Neural Architecture Searches
NHS: National Health Service
NHSE: NHS England
NIST: National Institute of Standards and Technology
OpenAI: AI research and deployment company
Oxford Nanopore: Tool for data integration
PacBio: Tool for data integration
PGx: Pharmacogenomics
PHM: Population Health Management
POCT: Point-of-Care Testing
PRS: Polygenic Risk Score
QA: Quality Assurance
RCPATH: Royal College of Pathologists
SC-VOC: Sickle Cell Vaso-Occlusive Crisis
SDG: Sustainable Development Goals
Sention: Tool for AI-Driven Analysis
TRE: Trusted Research Environment
UKAS: United Kingdom Accreditation Service
UNESCO: United Nations Educational, Scientific and Cultural Organization
VBC: Value-Based Care
WGS: Whole-Genome Sequencing
XAI: AI Assurances (Explainable Artificial Intelligence)
